# COVID-19 Pandemic: Epidemiology, Etiology, Conventional and Non-Conventional Therapies

**DOI:** 10.3390/ijerph17218155

**Published:** 2020-11-04

**Authors:** Abdur Rauf, Tareq Abu-Izneid, Ahmed Olatunde, Anees Ahmed Khalil, Fahad A. Alhumaydhi, Tabussam Tufail, Mohammad Ali Shariati, Maksim Rebezov, Zainab M. Almarhoon, Yahia N. Mabkhot, Abdulrhman Alsayari, Kannan R. R. Rengasamy

**Affiliations:** 1 Department of Chemistry, University of Swabi, Swabi, Anbar 23430, Khyber Pakhtunkhwa, Pakistan; mashaljcs@yahoo.com; 2 Pharmaceutical Sciences, College of Pharmacy, Al Ain University, Al Ain Campus 64141, UAE; tizneid@gmail.com; 3 Department of Biochemistry, Abubakar Tafawa Balewa University, Bauch 740272, Nigeria; olatundebch@gmail.com; 4 University Institute of Diet and Nutritional Sciences, Faculty of Allied Health Sciences, The University of Lahore, Lahore 54000, Pakistan; aneesahmedkhalil@gmail.com (A.A.K.); Tabussam.Tufail@dnsc.uol.edu.pk (T.T.); 5 Department of Medical Laboratories, College of Applied Medical Sciences, Qassim University, Buraydah 52571, Saudi Arabia; f.alhumaydhi@qu.edu.sa; 6 K.G. Razumovsky Moscow State University of Technologies and Management (the First Cossack University), 73 Zemlyanoy Val, 109004 Moscow, Russian; shariatymohammadali@gmail.com; 7 V.M. Gorbatov Federal Research Center for Food Systems of Russian Academy of Sciences, 109316 Moscow, Russian; rebezov@ya.ru; 8 Prokhorov General Physics Institute of the Russian Academy of Science, 119991 Moscow, Russian; 9 Department of Chemistry, College of Science, King Saud University, P.O. Box 2455, Riyadh 11451, Saudi Arabia; zalmarhoon@ksu.edu.sa; 10 Department of Pharmaceutical Chemistry, College of Pharmacy, King Khalid University, Abha 61421, Saudi Arabia; ygaber@kku.edu.sa; 11 Department of Pharmacognosy, College of Pharmacy, King Khalid University, Abha 61441, Saudi Arabia; alsayari@kku.edu.sa; 12 Institute of Research and Development, Duy Tan University, Da Nang 550000, Vietnam; 13 Faculty of Environment and Chemical Engineering, Duy Tan University, Da Nang 550000, Vietnam; 14 Indigenous Knowledge Systems Centre, Faculty of Natural and Agricultural Sciences, North-West University, Private Bag X2046, Mmabatho 2745, North West Province, South Africa

**Keywords:** epidemiology, Traditional Chinese Medicines, anti-viral, angiotensin-converting enzyme-2

## Abstract

Coronavirus disease 2019 (COVID-19), which reported in an outbreak in 2019 in Wuhan, Hubei province, China, is caused by the SARS-CoV-2 virus. The virus belongs to the beta-coronavirus class, along with the Middle East Respiratory Syndrome coronavirus and Severe Acute Respiratory Syndrome coronavirus. Interestingly, the virus binds with angiotensin-converting enzyme-2 found in host cells, through the spike (S) protein that exists on its surface. This binding causes the entry of the virus into cells of the host organism. The actual mechanism used by the COVID-19 virus to induce disease is still speculative. A total of 44,322,504 cases, a 1,173,189 death toll and 32,486,703 recovery cases have been reported in 217 countries globally as of 28 October 2020. Symptoms from the infection of the virus include chest pain, fever, fatigue, nausea, and others. Acute respiratory stress syndrome, arrhythmia, and shock are some of the chronic manifestations recorded in severe COVID-19. Transmission is majorly by individual-to-individual through coughing, sneezing, etc. The lack of knowledge regarding the mechanism of and immune response to the virus has posed a challenge in the development of a novel drug and vaccine. Currently, treatment of the disease involves the use of anti-viral medications such as lopinavir, remdesivir, and other drugs. These drugs show some efficacy in the management of COVID-19. Studies are still on-going for the development of an ideal and novel drug for treatment. In terms of natural product intervention, Traditional Chinese Medicines (TCM) have been employed to alleviate the clinical manifestation and severity of the disease and have shown some efficacy. This review presents an updated detailed overview of COVID-19 and the virus, concerning its structure, epidemiology, symptoms and transmission, immune responses, and current interventions, and highlights the potential of TCM. It is anticipated that this review will further add to the understanding of COVID-19 and the virus, hence opening new research perspectives.

## 1. Introduction

Coronavirus is an enveloped, positive single-strand RNA virus. It belongs to the Orthocoronavirinae subfamily, as the name suggests, whose members show characteristic “crown-like” spikes on their surfaces [1]. Coronavirus (CoV) is among the main pathogenic organisms that affect the respiratory system in humans. An ongoing outbreak of pneumonia associated with a novel coronavirus, called severe acute respiratory syndrome coronavirus 2 (SARS-CoV-2), was reported in Wuhan, Hubei province, China [2,3,4] in December 2019. On 11 February 2020, the novel virus began to cause pneumonia, and was named as coronavirus disease 2019 (COVID-19) by the World Health Organisation (WHO). In December 2019, the prevalence of the virus increased at an epidemic rate since its first occurrence in Wuhan [5]. Consistently, the international virus classification commission termed the virus as severe acute respiratory syndrome coronavirus-2 (SARS-CoV-2). The disease does not manifest initially as a chronic disease of the respiratory system. In previous decades, coronaviruses have caused Severe Acute Respiratory Syndrome (SARS) and Middle East Respiratory Syndrome (MERS) [6]. Currently, COVID-19 cases have been recorded globally. On the 1 March 2020, reports indicated that 79,968 individuals were infected with the disease of whom 41,681 were cured, and 2873 died [7]. On 31 January 2020, COVID-19 was presented by WHO as a Public Health Emergency of International Concern (PHEIC) [7]. 

**Figure 1:**
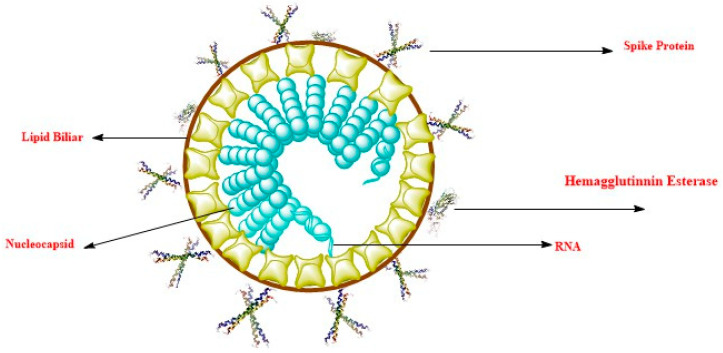
Structure of coronavirus virion showing the spike protein, membrane glycoprotein, lipid bilayer, nucleocapsid, envelope glycoprotein, RNA, and hemagglutinin esterase.

The coronavirus has a diameter of 80–120 nm and is single-stranded RNA. Four types of virus have been reported, which include α-coronavirus, β-coronavirus, δ-coronavirus, and γ-coronavirus [8,9]. Infection in humans is caused by six coronaviruses, and the 2019 novel coronavirus (SARS-CoV-2) is regarded as the seventh member of the coronavirus family to induce infection in humans [10]. The virus belongs to the beta coronavirus group like the MERS coronavirus (MERS-CoV) and SARS coronavirus (SARS-CoV) (which also cause disease in human). SARS and SARS-CoV-2 have approximately 79% genome sequence homology, and SARS-CoV-2 has a higher similarity to coronaviruses found in bats, causing SARS [8,11]. Intriguingly, many studies have revealed that SARS-CoV-2 binds with angiotensin-converting enzyme-2 (ACE-2), similarly to SARS-CoV; this is due to the similarity of the receptor-binding domain found on spike-proteins [12]. Coronaviruses use the spike (S) protein found on their surface to recognize and bind to specific receptors on host cell surfaces, resulting in virus entry to the cell of host and causing diseases [8]. SARS-CoV-2 forms a complex with ACE-2 more than ten times more significantly than SARS-CoV, greater than the threshold needed for the virus to cause disease, as revealed by the structure model procedure [13]. Comprehensive information on whether the 2019 nCoV causes disease in humans by the interaction of ACE-2 and its S-protein, how this interaction could serve as a means of disease transmission in humans, and how the virus causes damages to organs in patients remain unclear; thus, more detailed researches are necessitated [14]. The coronavirus virion is shown in Figure 1, revealing its various components. 

## 2. Pathogenesis

Individuals affected by COVID-19 present clinical symptoms such as non-productive cough, fatigue, fever, dyspnea, radiographic evidence of pneumonia and myalgia [15]. These clinical manifestations are similar to those of MERS coronavirus infection and SARS coronavirus [16]. Thus, though the pathogenesis of SARS-CoV-2 disease is not documented, the similar mechanistic action of MERS coronavirus and SARS coronavirus could provide valuable information on SARS-CoV-2’s disease pathogenesis to enable COVID-19 identification [7].

The entry of the virus into the cell of the host organism is determined by the S protein found on the virus [6]. The envelope S is constituted of glycoprotein binds to a specific receptor: CD209L (L-SIGN also called C-type lectin) for SARS coronavirus [17], ACE2 for SARS-CoV-2 [18] and SARS-CoV [19], and DPP4 for MERS coronavirus [20]. Initially, the movement of the virus into the cells of the host organism takes place through direct membrane fusion between SARS-CoV-2 and the host cell plasma membrane [21]. Significant proteolytic cleavage process takes place at the position (S2´) of the SARS coronavirus S protein, controlling the viral infectivity and binding to the membrane. An unusual activation of two-step furan for the fusion of the membrane is evolved in MERS coronavirus [22]. Apart from the fusion of the membrane, the clathrin-independent and -dependent SARS-CoV cell entry is regulated by endocytosis [23,24]. The virus’s genomic material is released into the cytosol after entry into the cells of the host organism. Then the genomic material is transcribed into two polyproteins and structural proteins, followed by the replication of the RNA [25]. The envelope glycoprotein that was newly formed is transported into the Golgi apparatus membrane, or endoplasmic reticulum, and the combination of the nucleocapsid protein with the genomic material results in the formation of the nucleocapsid. Subsequently, the viral materials enter the endoplasmic reticulum-Golgi intermediate compartment (ERGIC). The fusion of vesicles housing the viral materials results in their release [6].

After the virus entry into the cells of the host organism, the antigen presentation cells (APC) recognize the antigen of the virus. This is vital for the anti-viral immunity of the host cell. The major histocompatibility complex (MHC) in humans, or the human leukocyte antigen (HLA), are involved in the presentation of antigenic peptides, followed by the recognition of the peptide by virus-specific cytotoxic T lymphocytes (CTLs). Thus, knowledge of the presentation of SARS-CoV-2 will aid the understanding of coronavirus disease pathogenesis. Regrettably, information is still speculative, and only former documentation on MERS coronavirus and SARS coronavirus can be used. The MHC I molecules are largely responsible for the SARS coronavirus antigen presentation [7,26]. However, MHC II also partake in the presentation of antigen. Previous studies demonstrate that many polymorphisms of HLA are similar in SARS-CoV susceptibility, including HLA-DRB1*1202, HLA-B*0703, HLA-B*4601 [27], and HLA-Cw*0801 [28], while, HLA-A*0201, HLA-DR0301 and HLA-Cw1502 alleles are linked to SARS disease prevention [29]. In regards to MERS-CoV disease, molecules of MHC II, including HLA-DQB1*02:0 and HLA-DRB1*11:01, are associated with MERS coronavirus infection susceptibility [30]. However, mannose-binding lectin (MBL) gene polymorphisms linked to the presentation of antigen are linked to high chances of SARS coronavirus disease [31]. 

Studies have shown that ARDS (acute respiratory distress syndrome) is the leading cause of mortality in coronavirus disease and, out of 41 patients infected by this virus in the initial phase of the outbreak, 6 died from ARDS [15]. In MERS coronavirus, SARS coronavirus and SARS-CoV-2 diseases, ARDS is the prominent immunopathological feature [32](Xu et al., 2020). Cytokine storm is an essential mechanism of ARDS along with chronic unregulated systemic inflammatory stimulus, which is an outcome of the release of many of the pro-inflammatory markers including IFN-α, IL-1β, IL-12, IL-33, IL-18, IL-6, TGF β, TNF-α, etc. and chemokines like CXCL10, CXCL9, CXCL8, CCL2, CCL5, CCL3, etc., by immune effector cells [15,33,34,35]. Consistent with SARS coronavirus, patients with chronic MERS coronavirus disease display higher levels of IFN-α, IL-6 and CCL5, CXCL-10, and CXCL8 present in serum than those showing critical-moderate symptoms of the infection [36]. The cytokine storm stimulates an attack on the host body through the immune system leading to multiple organ failure and ARDS and eventually results in death in chronic cases of COVID-19, similar to cases seen in MERS coronavirus and SARS coronavirus diseases [32]. 

**Figure 2:**
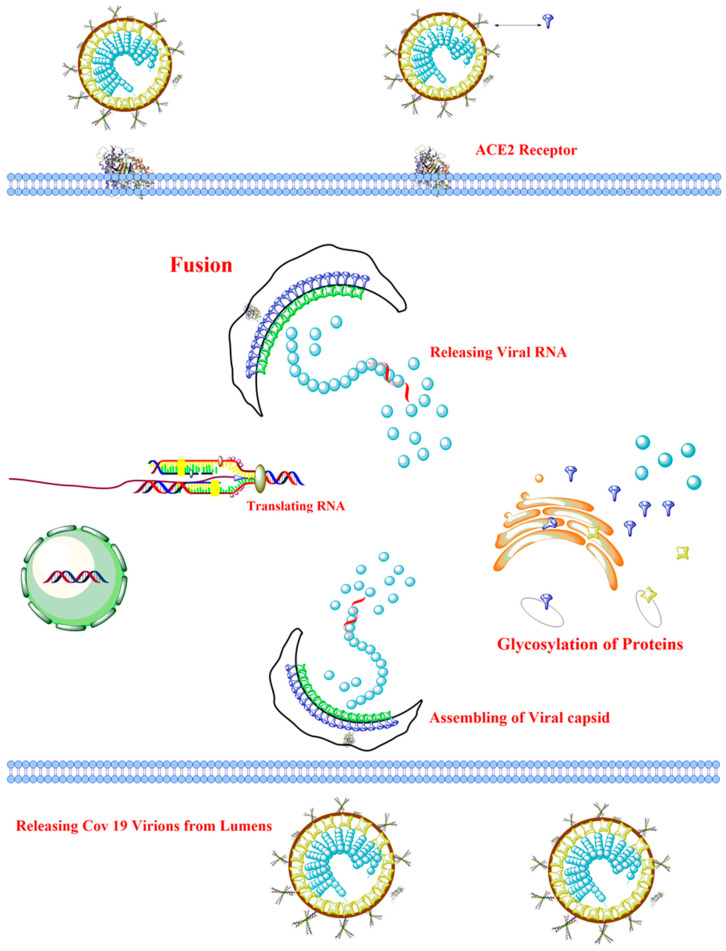
Scheme of Covid-19 pathogenicity mechanism.

The MERS coronavirus and SARS coronavirus employ several methods to prevent the immune response of the host cell. Pathogen-associated molecular patterns (PAMPs), which is an evolutionary conserved microbial substance, can be seen by pattern recognition receptors (PRRs). However, MERS coronavirus and SARS coronavirus can stimulate double-membrane vesicle synthesis. The synthesized vesicle possesses no PRRs, causing it to divide in the vesicles, hence preventing host determination of their double-stranded RNA [37]. IFN-β and IFN-α have demonstrated a protective role in disease caused by MERS coronavirus and SARS coronavirus; nevertheless, the pathways involving these cytokines is abrogated in infected experimental mice [38,39]. In MERS coronavirus disease, accessory protein 4a inhibits interferon (IFN) stimulation at melanoma differentiation-associated protein-5 (MDA-5) level induction, via the direct interaction with double-stranded RNA [40]. However, ORF5, ORF4a and ORF4b, and MERS-CoV membrane proteins block nuclear transport of regulatory factor 3 of the IFN and IFN β promoter stimulation [41]. Coronavirus can also influence antigen presentation. For instance, antigen presentation associated with gene expression is down-regulated after the infection of MERS-CoV [42]. Hence, management and specific drug formation are based on the impairment of immune evasion caused by coronavirus, resulting in COVID-19 [7]. Figure 2 depicts the mechanism of virus pathogenicity.

## 3. Epidemiology

Travel to Hubei Province in China was linked to the initial cases of coronavirus disease, but the increasing prevalence resulting from individual-to-individual transmission was documented both inside and outside of China [43]. In December 2019, more than 90% of reported coronavirus disease cases were from Hubei Province. However, by March 2020, the highest prevalence of COVID-19 was recorded in Italy, United States, Spain, France, Iran and Germany. In regards to data on coronavirus, the spread of COVID-19 is reported to be basically from individual-to-individual transmission by breathing droplets from coughing and sneezing via close contact [44]. Transmission of COVID-19 virus via contaminated areas or fomites following contact with the mouth, eye or nose may also take place. Symptomatic individuals are at the highest risk of infecting other individuals [45]. Limited information is available on viral shedding in individuals with asymptomatic features, but severely infected patients may present elevated viral shedding levels [46,47,48]. Recent patterns of the epidemiology of coronavirus disease in China demonstrate that the virus is highly infectious with sustained spread [49]. In the United States, human-to-human transmission was initially limited; however, currently, this has progressed to community spreading of the virus in several states [50]. Consistently, the recent basic reproduction number (R_0_) is estimated to be greater than 2.2; for every coronavirus disease case recorded in the population, more than 2 new cases are likely to be seen in the absence of adequate isolation [7,51,52]. The transmittance of SARS-CoV-2 has been observed to occur with a basic reproduction number (Ro) of 2.2–2.6. This means that on average each infected person can potentially spread SARS-CoV-2 to 2.2–2.6 other persons [11,53]. During the initial outbreak, in an epidemiological study of 425 coronavirus cases in Wuhan, 56% were male, and the median infected individual age was 59 years [7]. In 11 February 2020, 86.6% of infected individuals were between the ages of 30–70 years. The total fatality rate of the cases was 2.3%, and 80.9% of the documented cases were not severe. About 3.8% of the reported cases in hospital were health care workers, and 14.6% of these cases were either chronic or critical. A small number of cases (2.1%) has been documented for infants infected by the virus. Individuals severely infected by the coronavirus were found mostly among patients over 80 years of age, constituting about 14.8% of the total. For COVID-19 cases to be considered as chronic, the following features are met: oxygen saturation greater than, or equal to, 93%, respirations greater than, or equal to, 30 breaths/min, PaO2/FiO2 greater than 300, presence of more than 50% infiltration of the lungs within 24–48 h, or dyspnea. Individuals infected with the virus and at the critical stage are those patients with multiple organ impairment, septic shock and/or impaired respiration. These resulted in about 5% of the observed populace with 49.0% case casualty rate. The case fatality rate was 0.9% for infected individuals without comorbid conditions, while 10.5% was recorded for infected individuals with comorbidities such as cardiovascular disorders [54]. Death rates have been estimated to be 11–15% [15,55]. Case fatality rates outside of China were shown to range from 1.2–5.1%, while that of Hubei Province was shown to be 18% (95% confidence level (Cl): 11 to 18) [56]. Comparing other current epidemic diseases such as Ebola virus disease (39.53) or SARS (9.56), the mean symptomatic case fatality rate was around 4.2% for coronavirus disease and is lower than that of SARS (9.56) and Ebola virus disease (39.53). For the H1N1 influenza pandemic in 2009 and the influenza pandemic in 2017, about 220 more cases were recorded [57,58]. The 14 days incubation period duration proposed for coronavirus disease is based on the known incubation period for similar coronaviruses after first exposure [45]. The actual incubation duration is 5.2 days (95% Cl: 4.1 to 7.0); however, it can be between 2–14 days [7]. In about 22–33% of infected individuals, associated infections are recorded, and these may be higher in individuals with critical conditions [14,59].

## 4. Etiology 

Complete viral genome analysis reveals that the virus shares 88% sequence identity with two bat-derived severe acute respiratory syndromes (SARS)-like coronaviruses, but is more distant from the severe acute respiratory syndrome coronavirus (SARS-CoV) [60]. Hence, it was temporarily called 2019-novel coronavirus (SARS-CoV-2). Coronavirus is an enveloped and single-stranded ribonucleic acid named for its solar corona like appearance due to 9–12 nm-long surface spikes [61,62]. There are four major structural proteins encoded by the coronaviral genome on the envelope, one of which is the spike (S) protein that binds to the angiotensin-converting enzyme 2 (ACE2) receptor and mediates subsequent fusion between the envelope and host cell membranes to aid viral entry into the host cell [4,63]. On 11 February 2020, the Coronavirus Study Group (CSG) of the International Committee on Taxonomy of Viruses finally designated it as severe acute respiratory syndrome coronavirus 2 (SARS-CoV-2) based on phylogeny, taxonomy and established practice [64]. Soon after, WHO named the disease caused by this coronavirus as Coronavirus Disease 2019 (COVID-19) [32]. Based on current data, it seems that bats might initially host COVID-19, which might have been transmitted to humans via pangolin or other wild animals sold at the Huanan seafood market, with subsequent spread via human-to-human transmission [60]. 

## 5. COVID-19 Pandemic Around the World

As of 28 October 2020, cumulatively 44,322,504 confirmed cases and the death toll of 1,173,189 had been reported in 217 countries and territories globally.

### 5.1. Asia: First Reported Case to Updated Pandemic

In early December 2019, the outbreak of SARS-CoV-2-related pneumonia was firstly observed in Wuhan, China. This outbreak was declared as the sixth public health emergency by WHO and was later named as COVID-19 (coronavirus disease 2019). Specifically, in China to date, 85,868 cases have been reported out of which 4634 patients died, and 80,936 have been discharged after recovering from COVID-19 (Table 1). Present studies reveal COVID-19 respiratory symptoms as high body temperature, dry cough, fatigue, sore throat, runny nose, hypoxia, and dyspnea. These clinical manifestations were found to be quite similar to those observed during SARS (2003) and MERS (2012), strongly indicating transmission via droplets and physical contacts. Nonetheless, the extent of uncommon indications such as loose motion, vomiting, and stomach discomfort vary among different population groups, with initial and moderate outburst often associated with specific respiratory symptoms [64]. Increasing evidence from previous SARS studies suggests that biopsy specimens of SARS coronavirus (SARS-CoV) patients showed viral detection in stools and feces [65]. Both respiratory samples and viral nucleic acid of loose stool subsequently tested positive [32]. 

### 5.2. Europe: First Reported Case to Updated Pandemic 

The National Reference Centre confirmed that the first case in Europe was on the 24 January 2020 in Bordeaux, France [66]. As of 28 October 2020, details of reported confirmed COVID-19 cases in Europe are shown in Table 1. 

### 5.3. America: First Reported Case to Updated Pandemic 

The U.S. Centers for Disease Control and Prevention reported the first SARS-CoV-2 positive patient in the United States of America on the 20 January 2020 [67]. As of 28 October 2020, details of reported confirmed COVID-19 cases in the U.S. are shown in Table 1.

### 5.4. Africa: First Reported Case to Updated Pandemic 

SARS-CoV-2 was reported to have reached the continent Africa on 14 February 2020 in Egypt. To date (28 October 2020), the details of confirmed cases are shown in Table 1. 

### 5.5. Oceania: First Reported Case to Updated Pandemic 

The Victoria Health Authorities confirmed the first case of COVID-19 in Australia on 25 January 2020. Current updates regarding COVID-19 patients in Oceania are shown in Table 1.

## 6. Symptoms

The disease presentation of 2019 novel coronavirus (SARS-CoV-2) disease is similar to that of the Severe Acute Respiratory Syndrome (SARS) coronavirus. Clinical manifestations include dry cough, chest pain, fever, myalgia, dyspnea and fatigue [10,14,15]. Dizziness, abdominal pain, nausea, headache, vomiting and diarrhea are less common clinical presentations [68]. During the first outbreak of the disease in Wuhan, a reported 99 cases presented symptoms such as headache, dyspnea, lymphocytopenia abdominal pain, diarrhea, mucus production and hemoptysis. About 74 of the infected individuals presented bilateral pneumonia [53]. Similar features were recorded in non-pregnant and pregnant women [18,55].

Coronavirus infected individuals presented prominent upper respiratory tract manifestation including sneezing or sore throat, thus proposing that the virus might have higher affinity to harboring with the lower respiratory tract, and this is different from the Middle East Respiratory Syndrome coronavirus (MERS) and Severe Acute Respiratory Syndrome coronavirus diseases [15].

Acute Respiratory Stress Syndrome (ARDS), shock, critical cardiac injury, hypoxemia, arrhythmia and critical renal damage were chronic complications recorded in coronavirus infected individuals [15,53] and patients at this stage of the infection are taken to the intensive care unit [18]. In a study of 99 coronavirus infected individuals, about 17% presented ARDS, and from these patients, 11% of mortality recorded was from multiple organ failure [53] and the median time from the initial presentation to ARDS was eight days [14].

## 7. Transmission 

The role of Huanan Seafood Wholesale Market in spreading infection is speculative. Several first reports of SARS-CoV-2 disease were linked to the Huanan Wholesale Market, proposing that the coronavirus disease 2019 (COVID-2019) virus was transmitted from animals to humans [69]. Nevertheless, genome research has displayed scientific validation that the virus originated from another place before being carried to the market, where it was propagated rapidly from individual-to-individual [70]. Medical staff and family members have reported the presence of individual-to-individual transmission [71]. Individual-to-individual transmission was reported to take place by close contact with an infected individual. Transmission normally occurs through breathing droplets from the sneezing and coughing of an infected person. Fomites are also seen as a major route of transmission of this disease. SARS-CoV was discovered to remain on surfaces for about four days while other coronaviruses can be seen on surfaces for about nine days [72,73]. There is also some controversy whether transmission of the infection is asymptomatic or not. A study revealed that transmission is asymptomatic; however, it was eventually discovered that the scientist did not interview the infected individuals directly, who had shown some symptoms before transmitting the infection [74]. Currently, another researcher reported the disease transmission to be asymptomatic.

Notwithstanding, such research and other related works could be limited by mistakes in self-reported observations [46,69]. Work on infection properties are increasingly changing and vulnerable to selection bias. One study showed that the average incubation duration was 5.2 days, (95% confidence interval (95% Cl): 4.1 to 7.0) [7]. Nevertheless, definitions of cases largely depend on a 14-day window period [50]. Varying outcomes and interpretations were employed to determine the basic reproductive number (R_0_). The mean amount of infections from a single infected patient in a total population is measured by R_0_ [75]. Works from initial occurrence discovered R_0_ to be 2.7 for SARS [76] and 2.4 for H1N1 influenza in the 2009 pandemic [41]. A recent report discovered that R_0_ was as high as 3.28 [77]. It is vital to involve the role played by super spreaders, which may be largely responsible for the occurrence in huge congregations; however, these would not significantly affect the R_0_ value because R_0_ determines a mean value [78]. During the pre-pandemic or acute stage of the outbreak, R_0_ may vary [75]. 

A study was conducted of nine pregnant women who became infected with coronavirus at the late stage of pregnancy. The study revealed that the coronavirus disease did not present severe outcomes in pregnant women as observed in non-pregnant individuals. Consistently, no vertical transmission of the disease was observed for intrauterine disease [79]. In another study consisting of 138 individuals infected with the coronavirus, 41% of cases were through hospital-linked transmission [80]. In a similar study carried out in health care settings, the number of medical workers infected with COVID-19 diseases increased gradually with time. This was attributed to the high level of exposure of health care workers to a high level of the virus through the high number of infected individuals [69]. In 12 February, 2020, 441 cases of coronavirus disease were reported in twenty-four countries [81]. The first case through importation was revealed in Thailand on 13 January 2020 [82]. The transmission rate of COVID-19 is found to be higher compared to SARS and MERS mainly due to increased globalization. It has been found that the major difference between the current pandemic (COVID-19) and SARS is the rate at which this infection is spreading. In its early stage, as compared to SARS, COVID-19 is noted to be more asymptomatic [83]. Liu et al. [84] have reported R_o_ for SARS-CoV-2 in the range of 1.4 to 6.49, more than WHO estimates (1.4–2.5) for COVID-19 and than observed in case of SARS (2.2–3.6) [85]. Despite strong public awareness campaigns and interventional response, SARS-CoV-2 is spreading contagiously more than SARS-CoV-1 [84]. 

## 8. Immune System Response

The novel coronavirus (SARS-CoV-2) is a new coronavirus that has not previously been identified. Lack of knowledge regarding specific immune responses against SARS-CoV-2 is one of the main challenges in vaccine development. Current investigations on pathogenesis and initial infection site for SARS-CoV-2 are still under process. The majority of patients infected by SARS-CoV-2 have shown respiratory problems while, in some cases, pathology seems to target other organs like heart, brain, and colon. COVID-19 has an asymptomatic incubation period of 2 to 14 days after infection. Since December 2019, SARS-CoV-2 has resulted in an increased number of deaths worldwide. AfCoV-2 for ACE2 receptor has found to be 10 to 20 times higher compared to SARS, explaining its higher reproduction number [13]. Various studies regarding 2019-CoV have emphasized the complex role of antibody-dependent enhancement (ADE) in the pathogenesis of coronaviruses [86]. ADE of any disease is the major concern in developing vaccines and antibody therapeutics, as theoretically the mechanism that triggers antibody defense against the virus may potentially augment the rate of infection [87]. 

To date, clinical settings and earlier findings during SARS and MERS outbreaks have revealed conflicting effects of ADE to 2019-CoV disease. It could be inferred from the results of different studies on SARS and MERS that ADE may aggravate COVID-19. Earlier clinical trials have shown a safety risk, associated with ADE, of respiratory syncytial virus (RSV). Conflicting evidence regarding ADE (vaccine-induced) of SARS in different efficacy trials has raised possible safety concerns. Viral (SARS-CoV) replication decreased in Chinese macaques that were immunized with modified vaccinia Ankara (MVA) viral vector but also caused severe lung injury when compared to unvaccinated macaques [88]. 

Currently, there is no conclusive scientific evidence that suggests the occurrence of ADE in 2019-CoV infection. Hypothetical views are drawn depending upon previous reports of SARS and MERS coronavirus or some in-vitro investigations of COVID-19. Tan et al. [89] found delayed viral clearance and high infection severity in 2019-CoV patients that were strong responders for IgG antibody. Outcomes of this study hint towards ADE to COVID-19. Development of poor and/or non-neutralizing antibodies may cause ADE in the case of COVID-19 [90]. Another study conducted by Lv et al. [91] has shown poor cross-neutralization antibody response in SARS-CoV and 2019-CoV, suggesting the development of non-neutralizing antibodies that might play a role in ADE. As mentioned earlier, ADE has been noticed in RSV, SARS, measles, and MERS diseases and may pose a risk of ADE for 2019-CoV vaccines and antibody therapies. Nonetheless, strong clinical evidence is still required in establishing the role of ADE in 2019-CoV pathology. Conclusively, outcomes of efficacy and clinical trials will be helpful in understanding ADE in 2019-CoV disease [87]. 

### 8.1. Probable Innate Immune Responses 

Currently, there is only limited data regarding the host innate immune status of SARS-CoV-2-infected individuals. A report on 99 COVID-19 patients revealed elevated neutrophils (38%), serum IL-6 (52%) & CRP (84%) and a reduction in lymphocytes content (35%) [5]. Elevated neutrophils and reduced content of lymphocytes are associated with severity and mortality. Additionally, patients admitted to the intensive care unit showed increased levels of plasma innate cytokines (TNF-α, MCP-1, IP-10, and MIP-1A) suggesting the involvement of pro-inflammatory conditions in propagation and intensity of disease [15]. Similar elevation in pro-inflammatory cytokines was noticed in other coronaviruses infections (SARS & MERS) [92]. Effectual innate immune response to viral infections depends on the responses of IFN1 (interferon type 1) and its downstream cascade, which regulates viral replication and initiation of an efficient adaptive immune response. A host receptor is probably responsible for the entry of SARS-CoV-2, as ACE2 was for SARS-CoV-1 [5]. This proposed receptor (ACE2), vital for the entry of SARS-CoV-2, expresses in type 2 alveolar cells present in the lungs [10]. Keeping in view the similarities between SARS-CoV-1 & SARS-CoV-2, which share a match of 79% of their genomes and the same host entry receptors, might help understand immune system responses to SARS-CoV-2.

Usually, innate immune cells recognize viral invasion by detecting PAMPs (pathogen-associated molecular patterns). In the case of RNA based viruses like coronavirus, PAMPs (dsRNA) are detected by RIG-I (retinoic acid-inducible gene-I) or MDA-5 (melanoma differentiation-associated protein-5). This detection triggers the production of NF-κB (nuclear factor-κB) via signaling cascade followed by translocation of activated NF-κB into the nucleus. On the other hand, E3 ubiquitin kinases phosphorylate, both IRF3 (interferon regulatory factor-3) and IRF7 (interferon regulatory factor-7), on entry to the nucleus, initiate the expression of type I interferons (IFNs). Type I interferons (IFNs) bind at the IFNAR (IFNα/β-receptor) and activate the JAK-STAT (Janus Kinase-signal transducer and activator of transcription) signaling pathway. This activation results in phosphorylation of STAT1 & STAT2 due to JAK1 and tyrosine kinase-2 (TYK2) kinases and later these phosphorylated STAT1 & STAT2 bind with interferon regulatory factor-9 (IRF9). This complex enters the nucleus and starts the expression of ISGs (interferon-stimulated genes). This expression of pro-inflammatory cytokines, interferons, and IFN-stimulated genes collectively acts as an innate antiviral immune response, which helps in limiting viral replication in infected cells [6]. The same innate immune response to SARS-CoV-2 is likely to be expected keeping in view the similarities between SARS-CoV-1 & SARS-CoV-2, such as the 79% match of genome sequence and the same host entry receptors, i.e., ACE2. 

The genome of SARS-CoV-2 is comprised of some extra gene regions, and amino acid sequencing of certain probable proteins of SARS-CoV-2 has revealed similarity (68%) with SARS [64]. Hence, an in-depth sequencing of each gene region might be beneficial in predicting the possible inferences of SARS-CoV-2 with the innate immune response system of the host. Hypothetically, in order to evade the innate immune system, SARS-CoV-2 also uses a similar mechanism as adopted by SARS. 

### 8.2. Adaptive Immune Responses

Generally, during viral infections, Th1-type immune response is mainly responsible for adaptive immunity. Generation of the cytokine microenvironment due to antigen-presenting cells usually regulates the direction of T cell response. Overall, the adaptive immune response is coordinated by T helper cells, whereas cytotoxic T cells kill those cells that are infected by the virus. Humoral immunity, also known as antibody-mediated immunity, significantly limits the rate of infection and further prevents re-infection from the same virus. Reports reveal the presence of immunoglobulin G (IgG) and NAbs (neutralizing antibodies) even after two years of infection [93]. Immunoglobulin M (IgM) was observed at ninth day of infection followed by IgG by week two. Moreover, serum collected from COVID-19 patients was reported to neutralize the virus as examined in an in vitro assay, signifying humoral immunity to SARS-CoV-2 [5]. 

In SARS, the response of the T cell against this coronavirus has been analyzed extensively. A study of 128-convalescent plasma samples revealed that the adaptive immune response involved primarily cytotoxic T cells (CD8+ T cell) more so than T helper cells (CD4+T cell). Strong T cell responses correlate with elevated NAbs. They also reported an increased level of Th2 cytokines (IL-4, IL-5, IL-6, & IL-10) in the fatally classified group [94]. Since neutrophils exhibit a destructive role in all contagions, the beneficial role of Th17 in CoV-infections remains unclear. Current evidence suggests that Th1 response is critical in controlling the activity of SARS and MERS, and if further exploited this may be helpful against SARS-CoV-2. 

## 9. Systemic Effects of SARS-CoV-2 Virus

Biopsy and stool samples of confirmed SARS patient have revealed the presence of a virus in the gastrointestinal tract [95]. The first reported COVID-19 patient in America had a two-day history of vomiting and nausea on hospitalization; the patient passed a loose stool on the second day of admission [67]. SARS-CoV-2 was found positive in the loose stool and other respiratory samples collected from that patient [67]. Viral nucleic acid was found in the saliva of COVID-19 confirmed patients revealing the possibility of salivary gland infection [96]. The digestive system may be a potential route of COVID-19 infection, other than the respiratory system. Bioinformatics analysis conducted by Zhang et al. [97] demonstrated that the expression of ACE2 (angiotensin-converting enzyme 2) was elevated in the AT2 lung cells, upper esophagus, stratified epithelial cells, and absorptive enterocytes from the colon & ileum. 

Accordingly, the prevalence of liver-related abnormalities increased in patients suffering from SARS-CoV-2 which might be due to the systemic effect of COVID-19 and/or adverse effect of medicines administrated to the confirmed patients [98]. Liver biopsy of SARS-CoV-19 patients has revealed liver-related injuries such as steatosis [99]. Further, data from other articles have shown the elevated frequency of AKI (acute kidney injury) in SARS-CoV-2 confirmed patients. In COVID-19 patients, the prevalence of renal abnormalities was associated with an increased rate of mortality [62]. Expression of ACE2 and TMPRSSs (cellular transmembrane serine proteases) are found to be the main contributors to the entry of COVID-19 into host cells [100]. Comparative studies have shown that co-expression of ACE2-receptor and RMPRSSs-gene in examined kidney cells was nearly the same as that observed in lungs and colon. This expression suggests kidney as one of the targeted organs for COVID-19. Proximal straight tubule cells and podocytes were reported to be kidney host cells [101]. Injuries to podocyte due to bacterial and viral attacks have resulted in proteinuria [102]. According to recent data, 43.9% of COVID-19 confirmed patients having AKI resulted in proteinuria [62]. Furthermore, COVID-19 was detected in collected urine samples of SARS-CoV-2 infected patients [103]. Based on the findings of Pan et al. [101], it could be concluded that COVID-19 has a cytopathic effect on proximal straight tubule cells and podocytes, eventually causing AKI in COVID-19 patients. In order to prevent accidental infections, they also emphasized careful handling and disposal of urine samples of SARS-CoV-2 patients. 

According to Mao et al. [104], out of 214 studied COVID-19 patients, 36.4% showed neurological manifestations. Among these, 5.7%, 14.8%, & 19.3% of patients manifested acute cerebrovascular diseases, impaired consciousness, and skeletal muscle injury, respectively. Further, in neurological tissues, the expression of ACE2 suggests the presence of COVID-19 virus in the central nervous system [105]. The SARS-CoV-2 virus may enter the brain either through the cribriform plate and/or by post systemic circulatory distribution trailed by a lung infection. Neurological manifestations have revealed the loss of smell, involuntary breathing, and loss of taste in COVID-19 patients [106]. Zhou et al. [5] detected the presence of SARS-CoV-2 virus in cerebrospinal fluid (CSF). COVID-19 develops due to the entry of SARS-CoV-2 into the host cell via ACE2 receptors. Infection due to SARS-CoV-2 has resulted in AMI (acute myocardial injury) and chronic damage to the cardiovascular system. Five out of forty-one confirmed COVID-19 patients reported elevated (>28 pg ml^−1^) levels of hs-cTnI (high sensitivity cardiac troponin I) [15]. Accordingly, data of 36/138 COVID-19 confirmed patients treated in intensive care unit showed greater (11 pg ml^−1^) hs-cTnI levels as compared to patients that were not admitted to ICU (5.1 pg ml^−1^). They further suggested that patients in the severe category are more prone to develop acute myocardial injury [8]. AMI in COVID-19 patients may be due to the expression of ACE2 [107]. SARS-CoV-2 infects the host cells via ACE2 receptors and causes damage to lungs, CNS, heart, colon, kidneys, and eyes; hence, attention must be given when treating COVID-19 patients to its systemic effects. 

## 10. Probable Treatments and Precautionary Measures

### 10.1. Potential Treatments 

#### 10.1.1. Pharmacological Drugs

##### Lopinavir/Ritonavir

Lopinavir (LPV) is an antiretroviral drug, which is highly specific for the inhibition of HIV-1 (human immunodeficiency virus-1) protease enzyme. Usually, it is co-administrated with ritonavir (r), another antiretroviral drug that inhibits the activity of Cytochrome P450, in fixed-dose combinations for the treatment of HIV-1 virus. LPV is reported to suppress the replication of the virus by inhibiting the activity of the main protease of severe acute respiratory syndrome coronavirus-1 (SARS-CoV-1) [108]. Based on the results of administration of LPV/r against SARS-COV-1, its use for the treatment of SARS-CoV-2 has been included in Chinese COVID-19 guidelines. In antiviral therapies mentioned, LPV/r is prescribed, to be orally administrated as two capsules each time, twice a day, having dosage as Lopinavir: 200mg/ritonavir: 50mg/capsule [109]. In the United States, pediatricians are recommending the dosage as 10 mg kg^−1^ suspension to be administrated orally in patients having a weight range of 15 to 40 kg. Currently, ten different registered clinical trials are in progress in Hong Kong, Thailand, China, and Korea that are investigating the potential of Lopinavir/ritonavir alone and/or in combination with other available antiviral drugs or TCM (traditional Chinese medications) for treating SARS-CoV-2 [110]. 

A study conducted by Young et al. [111] on 18 (male: 9, female: 9) confirmed COVID-19 patients in four hospitals in Singapore revealed that for three out of five (60%) patients who were receiving oxygen therapy, the subjection of LPV/r showed a reduction in body fever and supplemental oxygen within three days of administration. Similarly, within two days administration of LPV/r, virus shedding in nasal swabs cleared in two out of five patients (40%), whereas, two out of five (40%) hospitalized confirmed cases of COVID-19 that were subjected to LPV/r resulted in respiratory failure [111]. According to another published case, a 54-year--old Korean man who confirmed positive for SARS-CoV-2 on 26 January in Myongji Hospital, Korea was administrated LPV/r twice a day at a dose of 400 mg/100 mg each time. In this patient, the total load of the virus started decreasing from the next day post administration of LPV/r. Significant improvement was observed in clinical symptoms, and reduction was found in viral loads of the patient during treatment with LPV/r. Further clinical insight into the effectiveness of LPV/r is suggested to validate the use of this antiviral drug in treating COVID-19 patients [112]. 

Similarly, a published case of a person infected with COVID-19 admitted to People’s Hospital in Wuwei was treated successfully with combination therapy. Along with other medications, this combination therapy contained LPV/r and was orally administered to the patient at a dose of 800 mg/200 mg daily. It was further reported that patients from other hospitals, whose clinical symptoms did not improve due to application of interferon and corticosteroid therapy, responded positively towards the additional LPV/r medication that they received in their hospital [113]. Data from other published cases in which patients were subjected to LPV 400 mg/r 100 mg, alone or in combination with IFN-α, could be helpful in recommending LPV/r as an antiviral remedy [114,115,116]. Furtorts revealed the use of LPV/r along with other antivirals such as oseltamivir & ganciclovir and anti-bacterials in the treatment of confirmed patients of COVID-19 in Wuhan [8,53]. 

On the other hand, Cao et al. [117] publicized the outcomes of an open-label randomized control trial in which they randomly assigned 99 confirmed COVID-19 patients to an LPV/r group and the remaining 100 COVID-19 patients to a group that was given standard care. The primary end-point was either time interval from randomization to discharge from the hospital or betterment of two points on a seven category ordinal scale. In terms of clinical improvement, no significant difference was noticed between LPV/r-treated and standard care groups. However, the 28th-day mortality was mathematically lesser in LPV/r-treated group as compared to the standard care group. Even so, the number of patients having a detectable viral load, examined at different time intervals, was the same in both groups. They were of the view that LPV/r treatment was not beneficial to confirmed COVID-19 patients and further trials are required to assess the effect of LPV/r on novel coronavirus pneumatic patients [117]. It is difficult to validate the use of LPV/r alone or with multi-therapy in the treatment of SARS-CoV-2. Further clinical trials are recommended in this context.

##### Remdesivir

Remdesivir (GS-5734) is an adenosine analog antiviral prodrug developed by Gilead Sciences to treat the outbreak (December 2013 to January 2016) of Ebola virus in Western Africa. The active form (GS-441524) of remdesivir reduces the production of viral RNA by binding to RNA dependent RNA polymerase. Other than treating Ebola virus patients, it possesses broad-spectrum antiviral properties against various RNA based viral (SARS-CoV & MERS-CoV) infections [6,118,119,120]. According to in vitro and in vivo studies by Sheahan et al. [121] remdesivir, along with interferon-β, showed more antiviral properties compared to LPV/r-interferon-β in MERS-CoV. Various other studies have also shown antiviral activity similar to that of remdesivir alone or in combination with other medications in treating SARS & MERS [121,122]. The selective nature of RDV (remdesivir) towards viral polymerases makes it less toxic and safer for human usage. In a human airway epithelial cell model, Sheahan et al. [123] have shown a broad-spectrum therapeutic index of remdesivir against zoonotic and epidemic coronaviruses. Most recently, in MERS infected mice, initiation of RDV resulted in a significant reduction of lung viral load, pulmonary hemorrhage, and weight loss [121]. Keeping in view the effectiveness of remdesivir against SARS-CoV-1 & MERS and the similarities between SARS-CoV-2 & SARS-CoV-1, in the current pandemic situation it may be a promising candidate for the treatment of COVID-19. Therefore, US-FDA has approved RDV for use in COVID-19 infections.

Remdesivir could be used as a therapeutic option in the treatment of SARS-CoV-19 patients. Wang et al. revealed in vitro activity (EC_90_: 1.76 μM; EC_50_: 0.77 μM) of RDV against COVID-19 in Vero E6 cells. Their initial results demonstrated that RDV inhibited viral infections significantly in Huh-7 cells (human liver cell line) that were sensitive to COVID-19 [124]. According to Holshue et al. [67] who reported the diagnosis, clinical management and treatment of the first case of COVID-19 in the US was with a patient having a travel history of recent return from Wuhan on 15 January 2020. This thirty-five-year-old male was confirmed as a COVID-19 patient on 20 January 2020 through real-time PCR. Keeping in mind the deterioration of patients’ condition, medical officials have opted for compassionate use of RDV. Intravenous administration of remdesivir was started from the evening of day 7 of hospitalization. Initiation of RDV showed no adverse effect in the patient, and further clinical improvements were noticed on day 8 of hospitalization followed by discontinuation of supplemental oxygen and improvement in oxygen saturation [67]. A cohort study comprising of 53 severe SARS-CoV-2 patients that received compassionate use of RDV showed improved clinical signs in 68% (36/53) patients [125]. 

Around the globe, the current pandemic condition requires an urgent and effective therapeutic approach for the treatment of COVID-19. Choy et al. [126] conducted an in vitro assessment of antiviral compounds (both earlier testified & presently under evaluation in clinical trials) on inhibition of coronavirus in COVID-19 patients. They evaluated antiviral effectiveness of RDV (EC_50_: 23.15 μM), emetine (EC_50_: 0.46 μM), LPV (EC_50_: 26.63 μM), and homorringtonine (EC_50_: 2.55 μM) against SARS-CoV-2 viral replication in Vero E6 cells. Other compounds currently under trial, i.e. favipiravir or ribavirin, demonstrated no inhibitory effect on the replication of virus at 100 μM concentration. The combined effect of remdesivir (6.25 μM) and emetine (0.195 μM) resulted in maximum (64.9%) reduction in viral load. Therefore, the combined therapeutic approach may be helpful in achieving effective clinical results [126]. On the other hand, out of the first 12 patients reported positive with SARS-CoV-2 in the United States, three patients received RDV for 4 to 12 days under compassionate use. It was observed that all three patients administrated RDV suffered from nausea, rectal bleeding, or vomiting along with elevated aminotransferase contents. Clinical improvement was noticed in all reported patients; nonetheless, Kujawski et al. were not able to evaluate the effectiveness of RDV due to the absence of any reference drug [127]. Hence, to authenticate the safety and clinical effect of RDV on the replication of SARS-CoV-2 virus in confirmed patients, various clinical trials are designed and in progress. In China, two registered clinical trials (Identifier: NCT04252664 & NCT04257656) designed to assess the safety of RDV in COVID-19 patients (mild/moderate & severe) are currently either suspended or terminated due to unavailability of confirmed cases. However, in the United States, four (NCT04292899; NCT04292730; NCT04280705: NCT04302766) different ongoing trials designated to assess the safety and effectiveness of remdesivir are recruiting confirmed COVID-19 patients (severe & moderate). Outcomes of clinical trials may be helpful in determining the effect of remdesivir on COVID-19 patients. 

##### Chloroquine and Hydroxychloroquine 

Both chloroquine and hydroxychloroquine are the most commonly prescribed antimalarial drugs possessing lysosomotropic properties. Compared to chloroquine, hydroxychloroquine is a less toxic (~40%) antimalarial drug and is made by introducing OH-group into chloroquine. Pharmacokinetic studies have revealed a wide distribution of the effects of these antimalarial drugs throughout the body, including the lungs. Due to their immunomodulating and anti-inflammatory characteristics, they have gained the current attention of scientists as a probable therapeutic agent to treat SARS-CoV-2. For this purpose, Wang et al. assessed in vitro activities of seven different antiviral drugs against COVID-19 in Vero E6 cells. They reported EC_50_ and EC_90_ values of chloroquine as 1.13 μM and 6.90 μM, respectively [124]. These findings were in accordance with earlier data reported by Colson et al. [128], who evaluated the inhibitory (EC_50_: 1 to 8.8 μM) action of chloroquine against SARS and MERS in different experimental cell lines. According to Chinese guidelines for SARS-CoV-2, two advisories have been issued for the use of chloroquine in patients according to their body weight. First is for individuals (18 to 65 years) having B.W. above 50 kg, to be orally administrated with chloroquine as 500 mg, twice a day for seven days. According to the second advisory, adults having B.W. below 50 kg must receive chloroquine (500 mg), twice a day for two days followed by once a day for next five days [109]. Similarly, according to emergency use authorization issued by the FDA, chloroquine (1 g on day 1 followed by 500 mg for 4–7 days on a daily basis) and hydroxychloroquine (800 mg on day 1 followed by 400 mg for 4–7 days on a daily basis) are permitted to be administered to COVID-19 patients weighing 50 kg or above. 

Recently, Gao et al. stated that various patients (>100) administrated chloroquine on admission to the hospital showed decreased exacerbated pneumonia, improved lung conditions and reduced viral load. No severe side effects due to the use of chloroquine for treatment of COVID-19 were noticed in patients, as mentioned above [129]. In vitro studies have demonstrated that chloroquine limits the viral replication of COVID-19. However, further clinical trials to assess the safety, usage dose, and efficacy of chloroquine are required with some urgency [130]. The entry of coronavirus into the cell via the endolysosomal pathway (Burkard et al., 2014) and the wide spectrum antiviral capacity of chloroquine against SARS could make it a therapy to treat COVID-19 patients in the absence of any other known treatment. While searching on clinicaltrials.gov, we currently found 113 registered trials that are using either chloroquine/hydroxychloroquine alone or with other therapeutics as their interventional plan against COVID-19. Through PBPK (pharmacology based pharmacokinetic) modeling, Yao et al. have assessed the potential of hydroxychloroquine and chloroquine against COVID-19. Their results of in vitro study (infected Vero cells) revealed that hydroxychloroquine possessed more antiviral potency when compared to chloroquine, their EC50 reported as 0.72 μM & 4.47 μM, respectively [131]. In vitro study conducted by Liu et al. (2020) examined the cytotoxicity of chloroquine and hydroxychloroquine in Vero E6 cells (ATCC-1586) via CCK8 analysis and reported values for CC_50_ as 273.20 μM and 246.50 μM, respectively. They also assessed the inhibitory activity of chloroquine and hydroxychloroquine against COVID-19 at various (0.01, 0.02, 0.2, & 0.8) MOIs (multiplicities of infection) and reported EC_50_ in the range of 2.71–7.36 μM & 4.51–12.96 μM for chloroquine and hydroxychloroquine, respectively. In a nutshell, the anti-COVID-19 activity of chloroquine was higher compared to that of hydroxychloroquine at examined MOIs [132]. 

In France, a total of 20 patients confirmed positive for COVID-19 were treated with hydroxychloroquine (60 mg/day) and, at day 6 after initiation of treatment, this resulted in a less average viral carriage as compared to the negative control (untreated patients). It was also reported that the addition of azithromycin, along with hydroxychloroquine, more effectively reduced the viral load when administered patients [133]. Furthermore, Gautret et al. analyzed the combined effect of treated hydroxychloroquine and azithromycin for at least three days in a cohort of 80 COVID-19 patients. Clinically, viral load in samples collected from the nasopharynx was negative for nearly 93% (day-8) of patients, whereas in 97.5% the respiratory samples collected at day-5 were reported as negative. They suggested that there is a need to hasten trials to validate the use of these drugs at a larger scale, keeping in mind the current pandemic situation [134]. A randomized clinical trial comprising of 62 SARS-CoV-2 patients demonstrated that the time for a reduction in body temperature and cough were significant in patients treated with hydroxychloroquine. As compared to the control group (54.8%), 25 out of 31 (80.6%) patients in the hydroxychloroquine treated group showed improved pneumonia conditions [55]. Despite the lack of clinical trials, all the outcomes of early treatment with chloroquine and hydroxychloroquine for SARS-CoV-2 around the globe are quite encouraging. Additionally, dosage and time of initiation for both antiviral drugs must be in agreement with the discussed data herein.

##### Tocilizumab

Tocilizumab is an immunosuppressant having an inhibitory action on both the soluble and membrane-bound IL-6 (interleukin-6). IL-6 is responsible for immunologic responses and symptoms in patients suffering from CRS (cytokine release syndrome). In 2010, the FDA approved this humanized monoclonal antibody against rheumatoid arthritis [135]. Elevated concentration of IL-6 was observed in severe COVID-19 patients and this hyper-inflammatory condition was related to high mortality in China [5]. The effectiveness of tocilizumab has been assessed in severe COVID-19 patients hospitalized in China. Body temperature normalized and all other clinical manifestations improved in severe COVID-19 patients who received tocilizumab (400 mg/daily) intravenously after hospitalization. Out of 21 patients receiving tocilizumab, 19 patients (90.5%) were discharged from hospital after an average hospitalization time of 13.5 days. These findings suggest the use of tocilizumab as an effectual treatment against COVID-19 [136]. 

In another study, 15 confirmed SARS-CoV-2 patients were subjected to immunotherapeutic agent *i.e.* tocilizumab. These patients were categorized depending upon their disease state into moderate (2/15), serious (6/15), and critical (7/15). An abnormal elevation of IL-6 reduced in ten patients treated with tocilizumab. According to the authors, the administration of tocilizumab may be an effective option for the treatment of SARS-CoV-2 patients having elevated IL-6 levels [137]. Fu et al. have reported that use of tocilizumab may reduce the incidence of cytokine storm by targeting the IL-6 pathways. Treatment with tocilizumab resulted in improved clinical signs in treated patients such as ameliorated respiratory functionality and decrease in elevated body temperature. Hence, they suggest the use of tocilizumab to treat and decrease the mortality of severe COVID-19 patients having elevated IL-6 levels [138]. Results of single-cell analysis have also highlighted the effectiveness of tocilizumab treatment in SARS-CoV-2 patients [139].

Chinese COVID-19 guidelines have permitted the use of tocilizumab in treating COVID-19 patients with elevated IL-6 levels. The recommended single dose for intravenous administration of tocilizumab is prescribed as 400 mg (4 to 8 mg kg^−1^ B.W.). A repeated dose of 400 mg could be given to patients for whom the first dose is ineffective. They have further suggested that no more than two doses be given and the maximum dose single dose must not exceed 800 mg [109]. To date, there are 22 registered trials in progress focused on the use of tocilizumab alone or in combination with other drugs to assess its effectiveness as a potential treatment for COVID-9 patients. It is necessary to conduct trials having a bigger sample size to look in depth at the benefits and any adverse effects associated with its use.

##### Nitazoxanide 

Pharmacologically, Nitazoxanide is considered a broad-spectrum antiviral/anti-parasitic medication used to treat various infections (viral, protozoal, & parasitic). In vitro antiviral activity of nitazoxanide has been noted as EC_50_ of 2.12 μM in Vero E6 cells [124]. Results of this study are in harmony with the findings of Rossignol [140], who in LLC-MK2 cells examined the antiviral activity of nitazoxanide and tizoxanide against MERS and reported EC_50_ values as 0.92 & 0.83 μM, respectively. The inhibition of viral load was noted via interference of the host-regulated pathways responsible for the replication of the virus, instead of virus-specific pathways [140]. Its antiviral potential has also been analyzed against influenza, and results revealed that nitazoxanide (600 mg) when orally administrated twice a day caused clinical improvement in patients [141]. In France, use of nitazoxanide is recommended by experts for the treatment of COVID-19 taking into account its in vitro antiviral properties. Currently, five trials (NCT04348409, NCT04343248, NCT04351347, NCT04341493, & NCT04345419) have been registered to assess the safety and effectiveness of nitazoxanide against novel CoV-2. Despite this, some experts have not recommended nitazoxanide for the treatment of COVID-19 [142]. Even though in vitro studies have shown encouraging antiviral activity of nitazoxanide, nevertheless detailed clinical trials are required to ensure effective dosage and safety.

##### Favipiravir (FVP) and Ivermectin

Favipiravir (FVP, a synthetic pro-drug) is an antiviral drug that is approved in Japan as a selective inhibitor of influenza virus. Keeping in mind its effective treatment against influenza virus, FVP is being considered as a potential candidate to treat COVID-19 patients. According to a randomized clinical trial, 116 and 120 patients were treated with FVP (Day 1: 1600 mg (twice) + Day 2–10: 600 mg (twice)) and Arbidol (Day 1–10: 200 mg (thrice)), respectively. As compared to Arbidol, the FVP treated group showed reduced latency to relief for cough and pyrexia. However, a non-significant improvement was seen in clinical recovery rate observed at day 7 in both groups [143]. Another study (single-centered) conducted to assess the influence of a cocktail therapy in the treatment of severe COVID-19 patients (thirteen) revealed the benefits of FVP administration [144]. A 58-year-old male, who had a kidney transplant two years ago and diagnosed positive for COVID-19, showed favorable results in the case of FVP treatment [145]. A meta-analysis conducted to assess the effectiveness and safety of Favipiravir against COVID-19 has reported clinical and radiological improvements in patients treated with FVP as compared to standard care. Nevertheless, non-significant differences were concluded for virus clearance and oxygen-support requirements [146]. Furthermore, in an open-label (comparative) control study, FPV treated COVID-19 patients (N: 35) had better viral clearance and CT-scan (chest) results as compared to lopinavir/ritonavir (N: 45) [147].

Ivermectin is a well-known FDA-approved drug used to treat parasitic infestations. Recently, this broad-spectrum antihelmintic drug has demonstrated anti-COVID-19 activity in an in-vitro study. Caly et al. [148] infected Vero/hSLAM cells with COVID-19 isolate (MOI: 0.1) for 2 h before applying ivermectin (5 μM). Harvesting of cell pellets and the supernatant was carried out at 0, 1, 2, & 3 days and replication of COVID-19 was assessed using RT-PCR (Reverse transcription-polymerase chain reaction). As compared to control (DMSO), at 24-hour, ivermectin resulted in a reduction of 93% and 99.8% in the supernatant and cell-associated viral RNA, respectively. They further reported an IC50 value for ivermectin at ~2 μM. At 48-hours, in comparison to control, a significant reduction (~5000-fold) was noticed in the viral RNA of ivermectin treated cells [148]. Further in-vivo and clinical trials are required to fully understand and authenticate the safety and effectiveness of FVP and ivermectin against COVID-19.

#### 10.1.2. Corticosteroids 

Corticosteroids are a class of steroid hormones used to reduce inflammatory responses in the lungs that might result in ARDS (acute respiratory distress syndrome). However, adverse effects associated with their use, such as the elevated risk of secondary infections and late viral clearance, may outweigh this benefit. The literature shows inconsistent evidence regarding the use of corticosteroids against SARS. Some investigators do not recommend corticosteroids as their use has reported either no positive effect or even a detrimental effect in some cases, while a study has also reported reduced mortality in a critical patient [110,149]. On the other hand, corticosteroids (methylprednisolone) treatment could be helpful for patients in which ARDS develops as reduced risk of mortality was observed in the treated group [150]. 

According to Chinese COVID-19 guidelines for diagnosis and treatment, methylprednisolone (1 to 2 mg kg^−1^ day^−1^), a corticosteroid, is permitted to be used for 3–5 days in the treatment of patients in which ARDS has developed. They also suggested in these guidelines that a high dose of this corticosteroid may lead to delayed viral clearance [109]. Given the benefits and risk associates with the use of this corticosteroid, the Chinese Thoracic Society recommended its dose as ≤0.5–1 mg per kg B.W. on a daily basis for not more than seven days [151]. So far, ten registered trials are aimed at evaluating the safety and effectiveness of different types of corticosteroid against COVID-19. As mentioned earlier, the literature contains diverse results regarding the use of corticosteroids against coronaviruses. Therefore, the results of randomized control trials are awaited eagerly to clarify their use with COVID-19 patients. 

#### 10.1.3. Convalescent Plasma Therapy

Convalescent plasma therapy involves the transfer of plasma from recovered patients of a disease to currently infected patients. The basic approach behind this therapy is that the antibodies present in convalescent patients might be helpful against virus and in boosting the innate immune system [152]. Plasma separated from the blood of SARS-CoV-2 recovered patients could be an alternative therapeutic option keeping in mind the escalated number of patients affected by this disease around the globe. Earlier findings regarding the use of convalescent plasma in SARS- & MERS-affected pneumatic patients have revealed a reduced rate of mortality [55,153]. This therapeutic approach may modulate immunopathology associated with viral diseases like COVID-19. A vigilant clinical evaluation might determine the effectiveness of this passive immunotherapy in reducing the deterioration and mortality rate of COVID-19 patients [154]. In comparison, authenticated vaccines are not available for COVID-19 to date, and until then convalescent plasma therapy might be considered as a potent approach for treatment [155]. In China, through passive antibody administration, decrease in viral load and death rate was noticed [156]. 

According to guidelines issued by the Chinese National Health Commission regarding the treatment of COVID-19 patients, use of convalescent plasma therapy is allowed in critically and severely ill patients [109]. Similarly, the FDA has also issued guidelines regarding the recommended use of convalescent plasma to treat COVID-19 patients under expanded access [157,158]. Four critical COVID-19 patients including a pregnant woman recovered from the disease after administration of convalescent plasma along with supportive care [159]. Effectiveness of administrated convalescent plasma therapy has also been assessed in a descriptive study comprising of six confirmed SARS-CoV-19 patients. No substantial side effects were noticed in all four COVID-19 patients transfused with antibody compatible convalescent plasma. In patients 1 & 5, this administration eliminated the viral load examined through throat swab. Further, prompt elevation in the content of anti-COVID-19 antibodies was noticed in patients 2 & 3. Observations from this study specify the effective role of antibody plasma therapy against COVID-19 [160]. 

An initial report regarding the administration of convalescent plasma to COVID-19 patients in Korea also confirms the findings above mentioned. The case of two COVID-19 patients subjected to convalescent plasma therapy revealed improved clinical manifestations [161]. Similarly, five COVID-19 patients on ventilators in Shenzhen Third People’s Hospital, China, who received convalescent plasma therapy showed normalization of body temperature and elevation in the arterial partial pressure of O2 and the fraction of inspired oxygen ratio. After the transfusion, viral count became negative within twelve days of its application [162]. A pilot study conducted by Duan et al. demonstrated that ten critically ill COVID-19 patients, when transfused with convalescent plasma (200 mL, once) resulted in increased oxygen saturation within three days of transfusion. Post transfusion, concentration of CRP decreased and an increase in lymphocyte content was also observed in these patients. Conclusively, they highlighted the importance of using this therapy to improve the clinical condition of COVID-19 patients [163]. While searching on clinicaltrials.gov, 29 trials are registered focusing on the use of Convalescent plasma alone or in combination with other treatments to evaluate effectiveness in COVID-19 patients. Finally, results of randomized clinical trials are awaited to determine the dose, time of administration, and effectiveness of Convalescent plasma against COVID-19. 

#### 10.1.4. Traditional Chinese Medicine 

In the absence of anti-COVID-19 specific vaccines and drugs, treatment through antivirals, antibiotics, convalescent plasma, and corticosteroids have been put into practice by various clinicians [164]. The World Health Organization have also stated “to date, there is no specific medicine recommended to prevent or treat SARS-CoV-2” [165]. For centuries, Traditional Chinese medicines (TCM) have been employed to curtail the clinical manifestations associated with several infectious diseases. Traditional beneficial use of TCM and lack of specific treatments in the current situation makes TCM a complementary therapy for SARS-CoV-2. Earlier reports highlighted the importance of traditional Chinese medicine against SARS disease. Traditional Chinese medicine also ameliorates the adverse effects linked with the use of conventional antiviral drugs [166,167,168]. All these reports suggest the use of traditional Chinese medicine as a potential candidate in the curtailment of COVID-19. 

Thirteen different naturally occurring compounds present in TCM have been assessed against COVID-19. A total of 125 herbs available in China was reported to have ≥2 of these 13 natural compounds. Twenty-six out of these 125 Chinese herbs are classified as herbs having the potential to treat respiratory infection due to virus. In vivo pharmacological studies conducted by [169] revealed that these 26 Chinese herbs regulated viral infection and inflammatory reactions. They concluded that these herbal plants might possess compounds with anti-COVID-19 properties [169]. A brief report published on treatment procedures adopted for four COVID-19 confirmed patients revealed that they were administrated with a Shufeng Jiedu Capsule along with other antiviral therapy (Kaletra^®^). In three out of these four patients, the clinical symptoms associated with COVID-19 pneumonia improved after initiation of the treatment [68]. To warrant the use of Shufeng Jiedu Capsule against COVID-19, studies are required revealing the effectiveness of TCM, as other antiviral treatments were also given to the patients in this report. Another study explains the therapeutic potential of Shuanghuanglian oral liquid against COVID-19 and suggests further clinical trials in this context [170]. 

The Chinese government has supported the use of traditional Chinese medications to eradicate the risks associated with the COVID-19 pandemic situation. According to official statements of 26 provinces, TCM is recommended along conventional treatments. Recommendations issued on 3 March 2020 by the National Health Commission & State Administration of Traditional Chinese Medicine also stated the use of TCM as a treatment protocol for COVID-19. A brief overview of the use of TCM for COVID-19 according to NHC recommendations (7th edition) is discussed in Table 2 [109]. According to Ren et al. [171], 60107 TCM-treated COVID-19 patients had been confirmed as of 17 February 2020. To date, six different trials have been registered at clinicaltrials.gov focused on evaluating the efficacy and safety of TCM in COVID-19. Further, Yang et al. report fifty registered trials in China aimed at assessing the effectiveness of TCM (self-made & commercial) for the treatment of COVID-19 patients [172]. 

## 11. Conclusions

Currently, the SARS-CoV-2 pandemic has caused the main challenge to experts in the field of medicine for the development of drug/vaccine. Concerning its transmission rate, scientists around the globe are working up to their best knowledge to develop effective anti-SARS-CoV-2 therapies as early as possible. To date, various antiviral drugs have demonstrated potential in treating COVID-19 infection. Literature reveals that pharmacological drugs (Lopinavir/ritonavir, remdesivir, Chloroquine, Hydroxychloroquine, Tocilizumab, Nitazoxanide, Favipiravir (FVP) and Ivermectin), Corticosteroids, Convalescent plasma therapy, and traditional Chinese medicines (TCMs) are among the therapies possessing clinical significance against COVID-19. Although these therapies have shown certain therapeutic effects still COVID-19 remains a serious concern. Therefore, it is need of the time to conduct further clinical trials on a large scale to authenticate the effectiveness and safety of mentioned therapeutics in this review.

## Figures and Tables

**Table 1:** Updated COVID-19 cases as of 28 October 2020

Regions	Total Cases	Total Deaths	Total Recovered	Total Active Cases
Asia	13,328,711	237,480	11,789,290	1,292,941
Europe	8,921,074	255,942	3,571,142	5,093,990
North America	10,822,292	346,631	7,204,179	3,271,482
South America	9,465,599	290,300	8,451,570	723,729
Africa	1,747,522	41,854	1,429,087	276,581
Oceania	36,585	967	31,776	3842

**Table 2:** Recommendations for treatment through Traditional Chinese Medicine for SARS-CoV-2-pneumonia [109].

Diagnosis	Stage (s)	Clinical Manifestations	Recommended Chinese Patent Medicine	Recommended Prescription	
				Name	Weight (Grams)
**Medical observation**		Fatigue and gastrointestinal discomfort	Huoxiang Zhengqi capsules (pills, liquid, or oral solution)		
		Fatigue and fever	Jinhua Qinggan granules, Lianhua Qingwen capsules (granules), Shufeng Jiedu capsules (granules), Fangfeng Tongsheng pills (granules)		
**Clinical treatment (confirmed cases)**	**Light, moderate and severe patients (Lung cleansing & detoxifying decoction)**			Ephedra	9 g
				Zhi gan cao (Baked Licorice root)	6 g
				xing-ren (Apricot seed)	9 g
				shi-gao (Gypsum-fried first)	15–30 g
				Guizhi (Cinnamon Twig)	9 g
				Zixie (Water Plantain Rhizome)	9 g
				Zhuling (mushroom, Polyporus umbellatus)	9 g
				Baizhu (Atractylodes macrocephalae Rhizome)	9 g
				Fu-ling	15 g
				chai-hu (Bupleurum)	16 g
				Huang Qin (root of Scutellaria baicalensis)	6 g
				Ban Xia (Pinellia ternata)	9 g
				Sheng Jiang (Fresh Ginger rhizome)	9 g
				Zi Wan (Aster root)	9 g
				Dong hua (Coltsfoot flower)	9 g
				She Gan (Belamcanda Rhizome)	9 g
				Xi Xin (Wild Ginger, Asarum)	6 g
				Shan Yao (Chinese Yam, Rhizoma Dioscoreae	12 g
				Zhi shi (Bitter orange)	6 g
				Chen-pi (Tangerine peel)	6 g
				Huo xiang (Agastache rugosa)	9 g
	**Mild cases (Cold dampness and stagnation lung syndrome)**	Fever, fatigue, sore body, cough, expectoration, chest tightness, suffocation, loss of appetite, nausea, vomiting, sticky stools. Tongue has thin fat tooth mark or is faint red, and the coating is white thick rot or white greasy and the pulse is moisten or slippery.		Mahuang	6 g
				Shigao	15 g
				Xingren	9 g
				Qianghuo	15 g
				Tinglizi	15 g
				Guanzhong	9 g
				Dilong	15 g
				Xuchangqing	15 g
				Huoxiang	15 g
				Peilan	9 g
				Cangzhu	15 g
				Fuling	45 g
				Baizhu	30 g
				Shanzha	9 g
				Shenqu	9 g
				Maiya	9 g
				Houpu	15 g
				Binlang	9 g
				Caoguo	9 g
				Shengjiang	15 g
	**Mild cases (Dampness and heat-accumulation lung syndrome)**	low or no fever, slight chills, fatigue, heavy head and body, muscle soreness, dry cough, low phlegm, sore throat, dry mouth, do not want to drink more, or accompanied by chest tightness, no sweat or sweating, Or vomiting and loss of appetite, diarrhea or sticky stool. The tongue is reddish, and the coating is white, thick and greasy or thin yellow, and the pulse is slippery or sloppy.		Binlang	10 g
				Caoguo	9 g
				Houpu	10 g
				Zhimu	10 g
				Huangqin	10 g
				Chaihu	10 g
				Chishao	10 g
				Lianqiao	15 g
				Qinghao	10 g
				Cangzhu	10 g
				Daqingye	10 g
				Gancao	5 g
	**Moderate cases (Dampness and stagnation lung syndrome)**	fever, low cough and sputum, or yellow sputum, suffocation, shortness of breath, bloating, and constipation. The tongue is dark red and fat; the coating is greasy or yellow and the pulse is slippery or stringy.		Mahuang	6 g
				Xingren	15 g
				Shigao	30 g
				Yiyiren	30 g
				Cangzhu	10 g
				Huoxiang	15 g
				Qinghao	12 g
				Huzhang	20 g
				Mabiancao	30 g
				Lugen	30 g
				Tinglizi	15 g
				Juhong	15 g
				Gancao	10 g
	**Moderate case (Cold dampness lung syndrome)**	low fever, low body temperature, or no heat, dry cough, low sputum, fatigue, chest tightness, nausea, or nausea. The tongue is pale or red, and the coating is white or greasy, and the veins are pulsating.		Cangzhu	15 g
				Chenpi	10 g
				Houpu	10 g
				Huoxiang	10 g
				Caoguo	6 g
				Mahuang	6 g
				Qianghuo	10 g
				Shengjiang	10 g
				Binlang	10 g
	**Severe cases (Plague poison and lung closing syndrome)**	fever, flushing, cough, yellowish phlegm, or blood in sputum, wheezing, shortness of breath, tiredness, fatigue, dryness and stickiness, nausea, food loss, poor stool, and short urination. Red tongue, yellow greasy coating, slippery pulses.		Mahuang	6 g
				Xingren	9 g
				Shigao	15 g
				Gancao	3 g
				Huoxiang	10 g
				Houpu	10 g
				Cangzhu	15 g
				Caoguo	10 g
				Banxia	9 g
				Fuling	15 g
				Dahuang	5 g
				Huangqi	10 g
				Tinglizi	10 g
				Chishao	10 g
	**Severe cases (syndrome of flaring heat in Qifen and Yingfen)**	Hot fever, thirst, shortness of breath, shortness of breath, blurred vision, or spotted rash, or vomiting blood, bleeding, or convulsions in the limbs. Tongue ridges have few or no moss, and the pulse sinks finely, or floats large and counts.	Xiyanping injection, Xuebijing injection, Reduning injection, Tanreqing injection, Xingnaojing injection.	Shigao	30–60 g
				Zhimu	30 g
				Dihuang	30–60 g
				Shuiniujiao	30 g
				Chishao	10 g
				Xuanshen	30 g
				Lianqiao	15 g
				Mudanpi	15 g
				Huanglian	6 g
				Zhuye	12 g
				Tinglizi	10 g
				Gancao	6 g
	**Critical cases (syndrome of inner blocking causing collapse)**	dyspnea, dyspnea, asthma or need mechanical ventilation, fainting, irritability, cold sweating, dark purple tongue, thick or dry moss, large floating roots.	Xuebijing injection, Reduning injection, Tanreqing injection, Xingnaojing injection, Shenfu injection, Shengmai injection, Shenmai injection.	Renshen	15 g
				Fuzi	10 g
				Shanzhuyu	15 g
				Delivered with Suhexiang Pill or Angong Niuhuang Pill	

## Abbreviations

SARS-CoV-2	2019 novel coronavirus
Cl	confidence level
COVID-19	coronavirus disease 2019
ACE-2	angiotensin-converting enzyme-2
ARDS	acute respiratory disease syndrome
Cl	confidence level
COVID-19	coronavirus disease 2019
HLA	human leukocyte antigen
IFN	interferon
IL	interleukin
MERS	Middle East Respiratory Syndrome
MERS-CoV	Middle East Respiratory Syndrome coronavirus
MHC	major histocompatibility complex
PRRs	pattern recognition receptors
R_0_	basic reproduction number
SARS	Severe Acute Respiratory Syndrome
SARS-CoV	Severe Acute Respiratory Syndrome coronavirus
SARS-CoV-2	severe acute respiratory syndrome coronavirus 2
TGF-β	transforming growth factor beta
TNF-α	tumour necrosis factor alpha
WHO	world health organization
